# Effect of cycloxygenase-2 silencing on the malignant biological behavior of MCF-7 breast cancer cells

**DOI:** 10.3892/ol.2014.2395

**Published:** 2014-07-30

**Authors:** SHENG YANG, HUI HAN

**Affiliations:** 1 Department of Oncology, The Union Hospital of Fujian Medical University, Fujian Provincial Key Laboratory of Translational Cancer Medicine, Fuzhou, Fujian 350001, P.R. China; 2 Department of Breast Surgery, The Union Hospital of Fujian Medical University, Fuzhou, Fujian 350001, P.R. China

**Keywords:** RNA interference, cyclooxygenase-2, breast cancer, proliferation, invasion

## Abstract

The aim of the present study was to investigate the effect of cyclooxygenase-2 (COX-2) silencing on the malignant biological behavior of MCF-7 breast cancer cells. COX-2 short hairpin RNA (shRNA) and unassociated sequences were synthesized and a shRNA lentiviral vector was constructed. The vector was transfected into MCF-7 breast cancer cells, in which clones with stable expression were screened out. The expression of COX-2 mRNA and protein was silenced using RNA interference (RNAi). Quantitative polymerase chain reaction, western blotting, a mononuclear cell direct cytotoxicity assay (MTT assay), a cell invasion assay and scratch tests were performed to investigate the downregulation of COX-2 mRNA and protein expression, the proliferative activity and growth rate of MCF-7 breast cancer cells, the glioblastoma multiforme (GBM) penetrating capacity, the cell movement and migratory capacity, and vascular endothelial growth factor (VEGF)-A and VEGF-C protein expression. The results revealed that the sequence-specific shRNA significantly downregulated the expression of COX-2 at the mRNA and protein levels. Furthermore, the downregulation of COX-2 expression markedly decreased the invasive and metastatic capacities of the cells, suppressed the proliferation, decreased the rate of growth, decreased the capacity of GBM penetration and migration, and decreased the protein expression of VEGF-A and VEGF-C, the two key factors that regulate tumor angiogenesis and lymphangiogenesis. In conclusion, the RNAi technique effectively silenced COX-2 gene expression and inhibited MCF-7 breast cancer cell proliferation, invasion and metastasis by decreasing VEGF-A and VEGF-C expression, which regulates tumor angiogenesis and lymphangiogenesis. Therefore, an RNAi technique that targets COX-2 presents a promising prospect for breast cancer gene therapy.

## Introduction

Previous studies have shown that cyclooxygenase-2 (COX-2) expression is enhanced in various solid tumors, including breast, lung and colorectal cancer, and is closely associated with tumor proliferation, invasion and metastasis, and thus, COX-2 is considered a promising target in antitumor gene therapy (1,2). As an important method for investigating gene function, the RNA interference (RNAi) technique is a rapid, economical and highly efficient technique for silencing gene expression (3,4). In the present study, a short hairpin RNA (shRNA) lentiviral vector was constructed and its effect on malignant biological behaviors, including proliferation, invasion and metastasis, of breast cancer cells was investigated. In addition, the function of COX-2 in the carcinogenesis and development of breast cancer was verified, which permitted further study regarding these mechanisms.

## Materials and methods

### Materials

MCF-7 breast cancer cell strains and 293T cells were purchased from the Shanghai Cell Resource Center of the Chinese Academy of Sciences (Shanghai, China). The pSPAX2, pMD2G and pLVX-shRNA1 vectors were purchased from Clontech Laboratories (Mountain View, CA, USA). The Plasmid Midi kit was purchased from Qiagen (Valencia, CA, USA). Opi-MEM, *Escherichia coli* DH5α and Taq DNA polymerase were purchased from Invitrogen Life Technologies (Carlsbad, CA, USA). T4 DNA ligase, *Bam*HI and *Eco*RI restriction enzymes were purchased from New England Biolabs (Ipswich, MA, USA). Liposome Lipfectamine 2000, Dulbecco’s modified Eagle’s medium (DMEM), fetal bovine serum (FBS) and Trypsin were purchased from Invitrogen Life Technologies. The gel extraction kit was purchased from Tiangen Biotech (Beijing) Co., Ltd. (Beijing, Japan). KOD high fidelity enzyme polymerase chain reaction (PCR) kit and Taq enzymes were purchased from Toyobo Co., Ltd. (Osaka, Japan). DNA ladder was purchased from Fermentas International, Inc., (Burlington, Canada). The Transwell chamber was purchased from Chemicon (Temecula, CA, USA).

### Cell culture

The MCF-7 breast cancer cells were placed in DMEM containing l0% FBS, incubated at 37°C in 5% CO_2_, and digested and passaged with 0.25% trypsin every 2–3 days. Cells at the logarithmic phase of growth were used for the experiments.

### Design and screening of COX-2 shRNA

The target sequences were designed according to the COX-2 mRNA sequence obtained from GenBank (http://www.ncbi.nlm.nih.gov/genbank) and the shRNA design principles. Three pairs of shRNA were designed to target COX-2 (Table I). The synthesis of the shRNA was carried out by Hanheng Biological Technology (Shanghai) Co., Ltd. (Shanghai, China). The shRNA was subsequently transfected into 293T cells according to the manufacturer’s instructions for Lipfectamine 2000. The transfection results were observed under a fluorescence microscope 24 h later. After 36 h, the cells were collected and the protein was extracted. The most efficient shRNA was selected according to the results of western blotting.

### Construction and transfection of COX-2-shRNA lentiviral vector

The most efficient pair of shRNA sequences were selected as the inference target. A double-stranded DNA fragment, with cohesive termini of the *Bam*HI and *Eco*RI restriction enzymes, and the hairpin sequence of 5′-CCATTCTCCTTGAAAGGACTTTTC AAGAGAAAGTCCTTTCAAGGAGAATGG-3′, was synthesized *in vitro*. The fragment was ligated into pGC-LV vectors and then transfected into *E. coli* DH5α. Following amplification and screening, positive clones were sequenced by Invitrogen Life Technologies, the plasmid was extracted and the COX-2-shRNA lentiviral vector was recombined. The MCF-7 breast cancer cells transfected with COX-2-shRNA lentiviral vector were defined as the knockdown group (COX-2-shRNA), the cells with the negative control sequences as the mock group and the cells with no sequence as the blank group.

### Total RNA extraction and quantitative PCR detection

Total RNA was extracted using TRIzol (Invitrogen Life Technologies) and reverse-transcribed into cDNA. The RNA was then detected by quantitative PCR. COX-2 and GAPDH primers (internal control) were synthesized by Hanheng Biological Technology (Shanghai) Co., Ltd. The following primer sequences were used: COX-2 forward, 5-CCCTTGGGTGTCAAAGGTAA-3′ and reverse, 5′-GCCCTCGCTTATGATCTGTC-3′; and GAPDH forward, 5′-AGAAAATCTGGCACCACACC-3′ and reverse, 5′-AGAGGCGTACAGGGATAGCA-3′. The reaction conditions for PCR were as follows: Pre-denaturation at 95°C for 15 sec, denaturation at 95°C for 5 sec and annealing at 60°C for 30 sec, for 45 cycles. The mixture was then denatured at 95°C for 1 min at the end of the PCR and cooled to 55°C, whereby the double strands of DNA were able to combine sufficiently. Between 55 and 95°C the light absorption value was recorded for 4 sec at every 0.5°C, and using these values a melting curve was generated. Quantitative analysis was performed using the ratio of the target gene to GAPDH. The data were analyzed using the 2^−ΔΔCt^ method.

### Analysis of protein expression by western blotting

Total protein was isolated 72 h after transfection. Protein quantification was performed using the bicinchoninic acid assay. The protein sample was normalized simultaneously. The sample load was 30 μg total protein per lane. Protein from 10% SDS-PAGE gel was transferred to a polyvinylidene difluoride membrane following electrophoresis. The protein was blocked with 5% skimmed dried milk at 4°C. Next, the primary rabbit monoclonal anti-COX-2 (1:500), anti-vascular endothelial growth factor (VEGF)-A (1:800), anti-VEGF-C (1:800) and anti-GAPDH (1:4,000) antibodies (Cell Signaling Technology, Inc., Danvers, MA, USA) were added and the mixture was incubated overnight at 4°C on a rocking platform. Subsequent to being washed, the membrane was added together with the horseradish peroxidase-conjugated secondary antibody (1:4,000) and incubated for 2 h. The membrane was then developed using an enhanced chemiluminescence system (Pierce Biotechnology, Inc., Rockford, IL, USA) and exposed to X-ray film. The gray scales were then scanned using ImageJ software (National Institutes of Health, Bethesda, MD, USA).

### Determination of cell proliferation by MTT assay

The MCF-7 breast cancer cells at the logarithmic phase of growth from each group were seeded into 96-well plates at 100 μl/well, at a density of 1×10^4^ cells/well. The plates were incubated at 37°C in an atmosphere of 5% CO_2,_ with saturated humidity, and an MTT assay was performed 2 to 72 h after incubation. Optical density (OD) values were detected at a wavelength of 570 nm using a microplate spectrophotometer (UV1700; Shimadzu Corporation, Kyoto, Japan). The mean value of five wells was the final OD value used. The cell proliferation curve was generated with time as the horizontal axis and OD value as the vertical axis. The suppression rate of proliferation of the breast cancer cells was calculated as follows: Suppression rate (%) = [(1 - OD value of the COX-2 - shRNA group) / OD value of blank group] × 100.

### Detection of the invasive capacity of breast cancer cells by cell invasion assay

Matrigel artificial substrate was layered in the Transwell chamber. Cell suspension (200 μl) containing 1×10^5^ MCF-7 breast cancer cells from each group was added to the upper chamber and 10% FBS DMEM medium was added to the lower chamber. The chamber was incubated at 37°C, in an atmosphere of 5% CO_2_ for 48 h to fix and stain the cells. Images were captured with an optical microscope (magnification, ×100). The numbers of the cells in the center and the surrounding five zones were counted and the average was identified as the number of cells penetrating through the plasma membrane. Each experiment was performed on three wells and the same experiment was performed in triplicate.

### Detection of the cell migratory capacity by scratch test

Horizontal lines were scratched across the wells at the back of the 96-well plate using a marker pen and at least two lines were scratched for each well. A total of ~5×10^4^ cells were added to each well. The next day, lines perpendicular to the horizontal lines were scratched with the head of a pipette. The cells were washed twice with phosphate-buffered saline, and the sloughing cells were removed. Next, serum-free medium was added into the wells and the cells were incubated for 24 h. Images were captured under a fluorescence microscope. The vertical distance of the inner face of the scratch zone was measured. The cell migrated number was calculated as follows: Cell migrated number (%) = (vertical distance of the inner face of the scratch zone prior to repair - vertical distance of the inner face of the scratch zone following repair) / vertical distance of the inner face of the scratch zone prior to repair × 100.

### Statistical analysis

Data were analyzed using SPSS version 16.0 (SPSS, Inc., Chicago, IL, USA). Quantitative data are expressed as the mean ± standard deviation. The differences between groups were analyzed by variance. P<0.05 was considered to indicate a statistically significant difference.

## Results

### Screening of the COX-2-shRNA lentiviral vector

**Figure 1:**
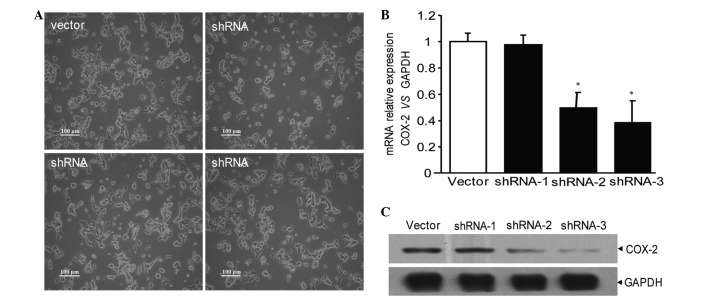
(A) Vector used was a no load virus. COX-2-shRNA-1, 2 and 3 represent three pairs of COX-2 shRNA, whose plasmids were effectively transfected into MCF-7 breast cancer cells (magnification, ×100). (B) Detection of COX-2 interference in each group by quantitative polymerase chain reaction. (C) Detection of COX-2 interference in each group by western blotting. COX-2, cyclooxygenase-2; shRNA, short hairpin RNA.

Vectors carrying plasmids with no sequence and COX-2-shRNA-1, 2 and 3 with three pairs of COX-2 shRNA, were effectively transfected into the MCF-7 breast cancer cells and emitted green fluorescence (Fig. 1A). According to western blotting and quantitative PCR detection, shRNA-1 exhibited no significant interference, shRNA-2 demonstrated partial interference and shRNA-3 exhibited the most significant interference (Fig. 1B and C). Therefore, the shRNA-3 sequence was used to recombine shRNA plasmid vectors. Next, the COX-2-shRNA plasmid was transfected into the MCF-7 breast cancer cells, then screened and amplified via G418 for further study.

### Effects of COX-2-shRNA on COX-2 mRNA in MCF-7 breast cancer cells

**Figure 2:**
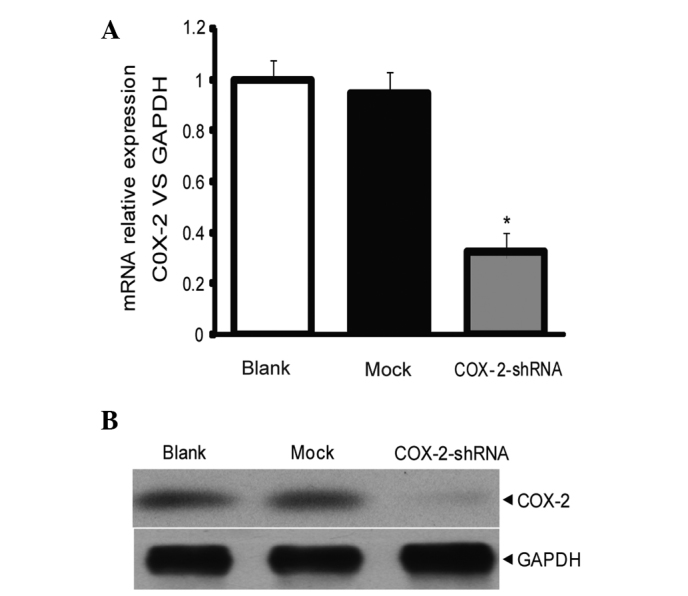
Expression of COX-2 (A) mRNA and (B) protein in MCF-7 breast cancer cells in the COX-2-shRNA group was significantly lower than that of the blank and mock groups. COX-2, cyclooxygenase-2; shRNA, short hairpin RNA.

As shown by quantitative PCR, the expression of COX-2 mRNA in the MCF-7 breast cancer cells of the COX-2-shRNA group was significantly lower than that of the blank and mock groups (P<0.05), with no significant difference identified between the mock and blank groups (Fig. 2A). Western blotting indicated that the expression of the COX-2 protein in the MCF-7 breast cancer cells of the COX-2-shRNA group was also significantly lower than that of the blank and mock groups (P<0.05), which was consistent with the results of the quantitative PCR (Fig. 2B).

### Effects of COX-2-shRNA on the proliferation of MCF-7 breast cancer cells

**Figure 3:**
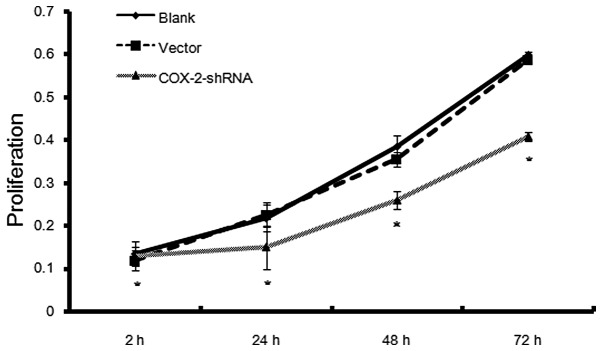
MTT assay revealed that the cell proliferation rates in the COX-2-shRNA group were significantly lower than that of the blank and mock groups. COX-2, cyclooxygenase-2; shRNA, short hairpin RNA.

A cell proliferation curve was generated based on the absorbance values of the MCF-7 breast cancer cells of the COX-2-shRNA, mock and blank groups, which were measured over 72 h. The initial absorbance values of the COX-2-shRNA, blank and mock groups were 0.0986±0.0076, 0.0994±0.0186 and 0.1037±0.0134, respectively, and no significant differences were identified (P>0.05). The absorbance values on day three for the COX-2-shRNA, blank and mock groups were 0.4949±0.0308, 0.6628±0.0245 and 0.6545±0.0155, respectively. No significant difference in cell proliferation rate was identified between the blank and mock groups (P>0.05), however, the cell proliferation rates of the COX-2-shRNA group were significantly lower than that of the other two groups (P<0.05). The suppression rates 24, 48 and 72 h after COX-2 interference were 24.47, 22.19 and 25.34%, respectively (Fig. 3).

### Changes in the invasive capacity of breast cancer cells following COX-2 interference

**Figure 4:**
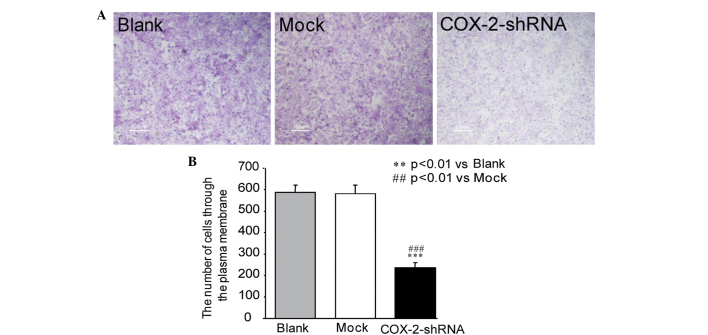
(A) Number of MCF-7 breast cancer cells penetrating the plasma membrane in the COX-2-shRNA group was significantly lower than that of the mock and blank groups 24 h after transfection (magnification, ×100). (B) The invasive capacity of the MCF-7 breast cancer cells was significantly decreased following transfection in the COX-2-shRNA group when compared with that of the blank and mock groups. COX-2, cyclooxygenase-2; shRNA, short hairpin RNA.

The Transwell assay demonstrated that the number of MCF-7 breast cancer cells penetrating through the plasma membrane in the COX-2-shRNA group was 235.5±25.6 at 24 h post-transfection, which was significantly lower than that of the blank (587.3±35.2) and mock (580.5±40.7) groups (P<0.05) (Fig. 4A). The results revealed that the invasive capacity of the MCF-7 breast cancer cells significantly decreased following transfection with COX-2-shRNA (Fig. 4B).

### Changes in the migratory capacity of breast cancer cells following COX-2 interference

**Figure 5:**
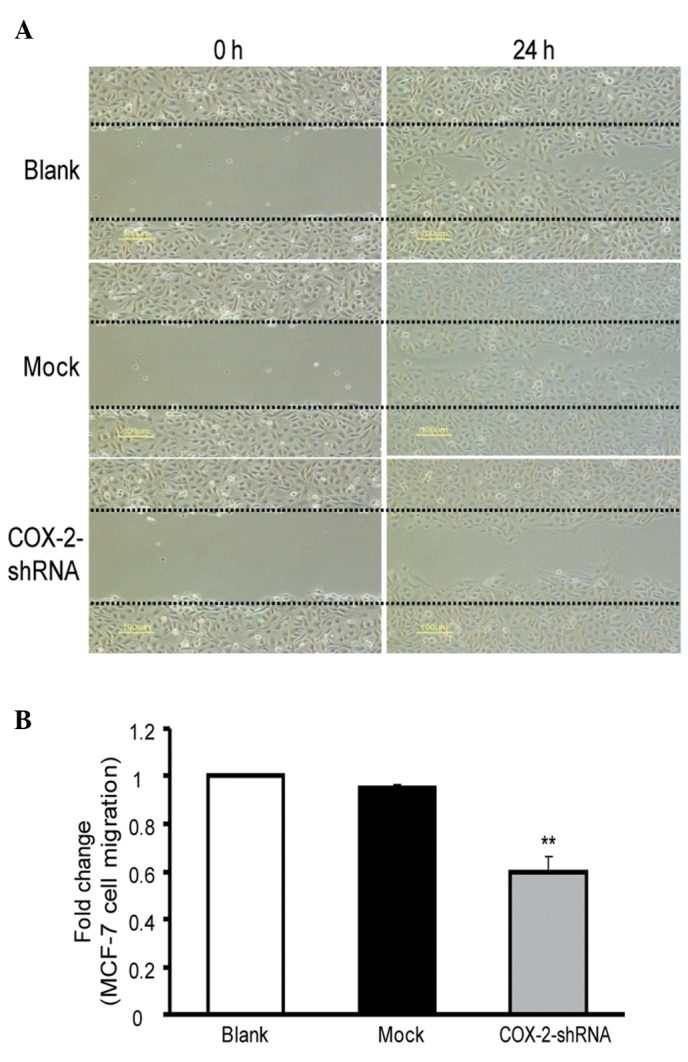
(A) Cell migration of MCF-7 breast cancer cells in the COX-2-shRNA group was significantly decreased (magnification, ×100). (B) Cell migration of MCF-7 breast cancer cells 24 h after transfection. COX-2, cyclooxygenase-2; shRNA, short hairpin RNA.

The cell migration of the MCF-7 breast cancer cells in the COX-2-shRNA group was markedly decreased (Fig. 5A). The cell migration at 24 h in the COX-2-shRNA group (59.6±7.0%) was significantly lower than that of the blank (100±0%) and mock (94.7±2.1%) groups, and the differences were statistically significant (P<0.05). However, no significant differences were identified between the blank and mock groups (P>0.05; Fig. 5B). These results revealed that the cell migration capacity of the MCF-7 breast cancer cells decreased significantly following transfection with COX-2-shRNA.

### VEGF-A and -C expression following COX-2 interference

**Figure 6:**
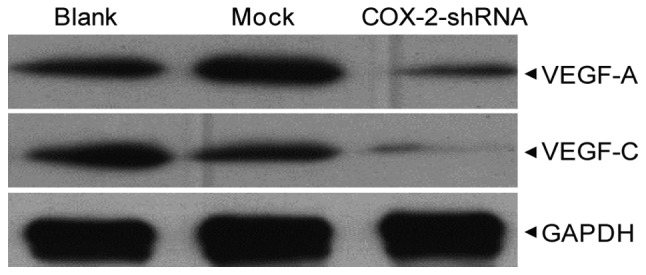
VEGF-A and -C protein expression of breast cancer MCF-7 cells in the COX-2-shRNA group was significantly lower than that of the blank and mock groups. COX-2, cyclooxygenase-2; VEGF, vascular endothelial growth factor; shRNA, short hairpin RNA.

Western blotting revealed that following COX-2-shRNA transfection, the expression of the VEGF-A and VEGF-C proteins in the breast cancer MCF-7 cells of the COX-2-shRNA group was significantly lower than that of the blank and mock groups (P<0.05), however, no significant differences were identified between the blank and mock groups (P>0.05) (Fig. 6).

## Discussion

Breast cancer has become a serious threat to the health of females worldwide (5). In China, the incidence of breast cancer is increasing at an annual rate of 3%, far surpassing lung cancer to become the malignant tumor with the fastest growing female mortality rate (6). The widely used comprehensive treatments of surgery, internal medical treatment and radiation therapy have yielded positive results, however, their efficacy is beginning to plateau (7). Studies of molecular tumor biology have identified that breast cancer is a genetic disease. The activation of oncogenes and the inactivation of tumor suppressor genes in the regulation of the cellular physiological processes leads not only to abnormal cell proliferation and differentiation, but also to defective apoptosis and drug resistance (8,9). Therefore, it is of great importance for the prevention and treatment of breast cancer to identify novel targets for breast cancer gene therapy (5,10). Previous studies have shown that COX-2 is abnormally expressed in various tumors, and is directly or indirectly involved in carcinogenesis and the development of tumors. The association of COX-2 with breast cancer has been an intense focus of previous studies (11,12).

COX-2 is an important rate-limiting enzyme in prostaglandin (PG) synthesis. At least two types of isoenzymes (COX-1 and COX-2) have been identified in mammals. COX-1 is a structural gene, which is expressed in normal tissue and cells and is involved in normal physiological functions. COX-2 is an inducible enzyme, which is undetectable in the majority of tissues under normal physiological conditions and which is only rapidly produced in specific cells when stimulated by mitogens, including cytokines, endotoxin, carcinogens and oncogenes (13,14). Previous studies have found that the carcinogenesis mechanism of COX-2 in breast cancer is complex and that COX-2 exhibits an important biological role in the proliferation, invasion and metastasis of breast cancer cells, as well as the regulation of the activity of relevant factors. Studies by Howe *et al* (15) and Singh and Lucci (16) have shown that COX-2 regulates PG synthesis, that its overexpression increases PG production, stimulating cell proliferation and promoting tumor formation, and that PGE2 and PGF2α, among others, stimulate Balb/C3T3 fibroblast mitosis together with epidermal growth factor (EGF). In addition, in the presence of EGF, PGE1 and PGE2 stimulate the growth of breast cells, and PGE2 functions as an epithelial cytokinin, directly stimulating the proliferation and growth of breast cells by increasing the levels of estrogen. Gabbert *et al* (17) revealed that tumor cells, when proliferating due to stimulation, increase the number of tumor cells with invasive potential and promote the division of cells surrounding the tumor margin, which creates the opportunity for tumor cell dissociation, and thus enhances the proliferation of infiltrating cells that form expansive tumor cell nests, to complete the process of invasion and metastasis. Takahashi *et al* (18) demonstrated that COX-2 enhances tumor proliferation and growth, as well as invasion and metastasis. Further studies by Sivula *et al* (19) and Bailey *et al* (20) have shown that by regulating the migration of tumor cells, enhancing the degradation of extracellular matrix and other activities, COX-2 increased the invasive capacity of cancer cells, further promoting the invasion of blood and lymphatic vessels, enhancing the transfer of cells to the lymph nodes and distant organs. The enhanced invasiveness is associated with the activity of tumor cell matrix metalloproteinase (MMP)-2, increased expression of MMP-1 mRNA, changes in urokinase plasminogen activator expression, cell adhesion molecule E-cadherin deficiency and the increased expression of hyaluronic acid receptor, CD44, on the cellular surface of tumor invasion and metastasis molecules. Takahashi *et al* (18) revealed that the breast cancer cell strain, Hs578T, with stable COX-2 expression exhibits an increase in the activity of MMP, thus increasing the capacity to digest the basement membrane, which provides more direct evidence for the involvement of COX-2 in cancer cell invasion. These results were consistent with the study by Hiraga *et al* (21). Tumor proliferation and growth, resistance to apoptosis and invasion and metastasis are closely associated with the formation of tumor blood and lymphatic vessels. VEGF and COX-2 exhibit multi-channel connections at the gene and protein expression levels. As demonstrated by Musto (22), colocalizations are frequently identified between the VEGF-3 and COX-2 genes, which indicates that a mechanism exists within the tumor cells that controls the expression of the two genes. Pai *et al* (23) used molecular biology to demonstrate that VEGF induces the expression of COX-2, and stabilizes COX-2 mRNA and protein expression via the COX-2 promoter, GATA-related locus, in vascular endothelial cells. In addition, it is hypothesized that the VEGF-induced increase in COX-2 occurs via the activation of p38 mitogen-activated protein kinase and c-Jun N-terminal kinase factor signaling. It is generally accepted that COX-2 is involved in the formation of tumor blood vessels. COX-2 significantly promotes the generation of angiogenic factors, including VEGF, basic fibroblast growth factor, transforming growth factor-1, platelet-derived growth factor and endothelin-1. Furthermore, Liu *et al* (24) and Uefuji *et al* (25) demonstrated that COX-2 increases VEGF-A expression in tumors and that COX-2 inhibition suppresses VEGF-A expression. In addition, Bamba *et al* (26) revealed that COX-2 acts on the associated receptors by promoting the synthesis of PGs, including PEG2 and 15-deoxy-PG J2, or induces the increase of VEGF-A-based angiogenesis factor expression by entering the nucleus directly via nuclear receptors to induce the formation of tumor blood vessels. As a member of the VEGF family, VEGF-C was the first lymphangiogenesis factor to be identified. It induces proliferation and migration of lymphatic endothelial cells and promotes lymphatic extension and nascent lymphatic sinus growth via the MEK/ERK and PI32 kinase/Akt pathways following binding to the VEGFR-3 receptor. Furthermore, in tumor lymphangiogenesis, VEGF-C induces internal and surrounding lymphangiogenesis, and promotes the growth and metastasis of lymphatic tumors (27). Su *et al* (28) revealed that COX-2 and VEGF-C expression in a human tumor cell line showed that VEGF-C was significantly higher in cell lines that overexpress COX-2, and further studies showed that COX-2 may increase VEGF-C expression via EP1 and human epidermal growth factor receptor 2 to promote tumor lymphangiogenesis and lymph node metastasis. Therefore, inhibition of the COX-2 gene may inhibit the generation of tumor blood and lymphatic vessels and suppress tumor invasion and metastasis, as well as proliferation and growth.

RNAi is the most effective antisense technique at present, originating from a hereditary phenomenon widely existing in flora and fauna, and serving as a protective mechanism against the gene instability caused by viral infection and insertion mutations. The technique specifically induces the degradation of target mRNA using double-stranded siRNA. In comparison to other gene knockout techniques, RNAi exhibits high efficiency, stability, specificity, hereditability and transmissibility, and therefore is significant in the research of gene function and in tumor gene therapy (29,30). In the present study, quantitative PCR and western blotting demonstrated that COX-2-shRNA effectively suppressed the expression of COX-2 mRNA and protein in MCF-7 breast cancer cells. In addition, the MTT assay revealed that the COX-2-shRNA sequence altered the proliferation and growth of the cells. In particular, the suppression rate of the MCF-7 breast cancer cells was 24.47, 22.19 and 25.34%, 24, 48, and 72 h after COX-2 interference, respectively. The present study demonstrated the importance of COX-2 in maintaining and promoting the proliferation and growth of breast cancer cells at the mRNA and protein levels. Following the transfection of COX-2-shRNA into the breast cancer cells, the invasion and migration capacities were significantly altered, as shown by the markedly decreased cell membrane-penetrating capacity and erasion trace repair rates. All data demonstrated the importance of COX-2 in the invasion and migration of breast cancer cells. In addition, as shown in the literature, in the COX-2-shRNA group, the mRNA and protein expression was reduced significantly and the protein expression of VEGF-A and VEGF-C was also markedly decreased when compared with that of the other groups. Therefore, COX-2 downregulation via RNAi is one of the predominant mechanisms that inhibit the malignant biological behaviors of breast cancer by reducing the activity of VEGF-A and VEGF-C, which promote tumor angiogenesis and lymphangiogenesis. Numerous studies have demonstrated that COX-2 inhibitors exhibit a positive effect against breast cancer. McCormick *et al* (31) found that indomethacin can reduce the incidence of breast cancer and tumors induced by dimethyl-benzanthracene (DMBA). Harris *et al* (32) used celecoxib in a DMBA-induced breast cancer model and found that the drug markedly delayed the occurrence of tumors, and that this was more effective when compared with ketoprofen. Furthermore, Nakatsugi *et al* (33) demonstrated that nimesulide, another COX-2 inhibitor, decreases the incidence of breast cancer by 28% in a rat model. Harris *et al* (34) revealed that the administration of non-steroidal anti-inflammatory drugs (NSAIDs) for between five and nine years reduces the incidence of breast cancer by 21%, and that administration for >10 years reduces the incidence by 28%. In addition, Khuder and Mutgi (35) recorded that NSAIDs reduced the risk of breast cancer, with a coefficient of relative risk factor of 0.8 (95% confidence interval, 0.75–0.89).

In conclusion, the downregulation of COX-2 gene expression suppresses the malignant biological behavior of breast cancer cells. Further studies investigating the association between COX-2 and breast cancer may identify methods of regulating COX-2 expression to prevent and control breast cancer, thus presenting novel approaches for breast cancer prevention and treatment (36).

## Figures and Tables

**Table I:** shRNA sequences specific to COX-2.

shRNA number	Sequence
COX-2 shRNA-1	GCTGAATTTAACACCCTCTAT
COX-2 shRNA-2	GCAGATGAAATACCAGTCTTT
COX-2 shRNA-3	CCATTCTCCTTGAAAGGACTT

shRNA, short hairpin RNA; COX-2, cyclooxygenase 2.
